# Weekly Time Course of Neuro-Muscular Adaptation to Intensive Strength Training

**DOI:** 10.3389/fphys.2017.00329

**Published:** 2017-06-08

**Authors:** Niklas Brown, Dieter Bubeck, Daniel F. B. Haeufle, Johannes Weickenmeier, Ellen Kuhl, Wilfried Alt, Syn Schmitt

**Affiliations:** ^1^Department of Biomechanics and Sports Biology, Institute of Sports and Movement Science, University of StuttgartStuttgart, Germany; ^2^Multi-Level Modeling in Motor Control and Rehabilitation Robotics, Hertie Institute for Clinical Brain Research, Eberhard-Karls Universität TübingenTübingen, Germany; ^3^SC SimTech—Stuttgart Centre for Simulation Sciences, University of StuttgartStuttgart, Germany; ^4^Department of Mechanical Engineering, Stanford UniversityStanford, CA, United States; ^5^Departments of Mechanical Engineering and Bioengineering, Stanford UniversityStanford, CA, United States; ^6^Biomechanics and Biorobotics, University of StuttgartStuttgart, Germany

**Keywords:** virtual gym, muscle volume, interpolated twitch, resistance training, modeling, simulation

## Abstract

Detailed description of the time course of muscular adaptation is rarely found in literature. Thus, models of muscular adaptation are difficult to validate since no detailed data of adaptation are available. In this article, as an initial step toward a detailed description and analysis of muscular adaptation, we provide a case report of 8 weeks of intense strength training with two active, male participants. Muscular adaptations were analyzed on a morphological level with MRI scans of the right quadriceps muscle and the calculation of muscle volume, on a voluntary strength level by isometric voluntary contractions with doublet stimulation (interpolated twitch technique) and on a non-voluntary level by resting twitch torques. Further, training volume and isokinetic power were closely monitored during the training phase. Data were analyzed weekly for 1 week prior to training, pre-training, 8 weeks of training and 2 weeks of detraining (no strength training). Results show a very individual adaptation to the intense strength training protocol. While training volume and isokinetic power increased linearly during the training phase, resting twitch parameters decreased for both participants after the first week of training and stayed below baseline until de-training. Voluntary activation level showed an increase in the first 4 weeks of training, while maximum voluntary contraction showed only little increase compared to baseline. Muscle volume increased for both subjects. Especially training status seemed to influence the acute reaction to intense strength training. Fatigue had a major influence on performance and could only be overcome by one participant. The results give a first detailed insight into muscular adaptation to intense strength training on various levels, providing a basis of data for a validation of muscle fatigue and adaptation models.

## Introduction

Homeostasis is a basic principle of biology that was first mentioned by Cannon (1926). It describes the process of a biological organism to keep its settled, internal status, according to the respective environmental condition. That is, it controls the remaining state, e.g., blood temperature, as long as no unusual, external event perturbs the system. Unusual perturbations can lead to changes in the organism's status (Brooks and Myburgh, 2014). For skeletal muscle, unusual external perturbations are, for example, intensive strength training or severe muscular underloading, as in subjects with no or dramatically less physical activity from extended bed rest or space flight. In such cases, homeostasis triggers adaptation processes such that the organism is able to deal with the varied environmental conditions. Biologically, the known adaptation processes in skeletal muscle are hypertrophy, the increase of muscle mass through cell growth, and atrophy, the decrease of muscle mass. Predicting the time course of muscular adaptation is of great interest in research in order to better understand biological aging, to optimize space flight training protocols, to establish counter measures and preventional training procedures, or to optimize pure muscular training protocols.

To describe the basic processes and time scales of adaptation, models of training and training effects have been published for about 40 years; for example, the theory of supercompensation (Jakowlew, 1974) or later the fitness-fatigue model by Zatsiorsky (1995). Although these models predict human performance capabilities on a very course scale, on the organism level, they are already partly able to predict the time scales of training effects. More recently, a numerical model was developed taking detailed physiological considerations of fatigue into account (Eriksson et al., 2016). All of these models allow for a basic initial understanding of the adaptation, which can help in optimizing, for example, athletes' performance training. However, none of these models can actually describe a subject-specific time course of adaptation, especially on the level of the individual organ or muscle.

It is known, that unphysiological stress on a muscle, as observed in chronic mechanical loading during physical exercise, can cause adaptation of various muscular mechanisms on different scales (Crewther et al., 2005, 2006a,b; Toigo and Boutellier, 2006). It is reported, that the muscle fiber length is adapted by a change in the number of sarcomeres, a change in the cross-sectional area, or a change in the muscle volume (Wisdom et al., 2014). Recently, a first model studying muscular adaptation on the organ level has been published, predicting the fiber length adaptation to changes in muscle strain (Wisdom et al., 2014; Zöllner et al., 2015). This approach even allows for a spatial resolution of muscle adaptation within a single muscle belly.

Although the overall effects of training are described in many publications (Crewther et al., 2005, 2006a,b; Kraemer et al., 2009), the detailed time course of adaptation to heavy strength training is not yet clear since many study designs lack a continuous monitoring or too large unmonitored phases in-between (Radaelli et al., 2014). In contrast to previous findings (Phillips, 2000; Blazevich et al., 2007), recent research on the time course of hypertrophic adaptation show that muscle growth already occurs after short periods of training (DeFreitas et al., 2011; Baroni et al., 2013). These authors analyzed untrained individuals, looking at muscle cross-sectional area and maximum isometric voluntary contraction. In untrained women, Stock et al. (2016) also found rapid increases in strength and morphological parameters such as thigh lean mass and cross-sectional area.

To generate a detailed dataset for further model development and validation, it is necessary to document training and corresponding adaptation at an appropriate level of detail. The purpose of this pilot study is to report comprehensive results of two exemplary cases of muscular adaptation to a subject-spefic training protocol. We recorded in detail the training stimulus and the time course of morphological and functional muscular adaptation, i.e., muscle volume, resting twitch torques, voluntary activation, and maximum strength. These properties were measured every week for 12 weeks, including pre-test, 8 weeks of training, and detraining. With this approach we aim to provide a well-controlled data set for a better understanding of adaptation and a basis for the validation of training effect models.

## Methods

### Participants

Two active, healthy men (subjects D and M; age: 24 years and 23 years, resp.; weight: 83 kg and 65 kg, resp.; height: 186 cm and 175 cm, resp.; strength training experience: 0 year and 1.5 years, resp.) volunteered to participate in this study. Both participants gave written and informed consent to the experimental procedure. The measurements used were approved by the local ethics committee. Participants were asked to keep their normal activity level and keep their diet unchanged during the study. Participant D reported very few training experience, whereas participant M had experience in heavy strength training.

### Study design

The study was designed as a longitudinal case study with a total of 8 weeks of intensive strength training followed by a 2-week long detraining phase. The subjects trained on Mondays, Wednesdays, and Fridays. Measurements took place every Wednesday. One week before the first training, initial measurements were conducted to establish a baseline level of the tested parameters. In total, twelve data points were collected for each subject: two sets prior to training, eight sets during the 8-week long training period, and two sets during detraining.

### Training

At the beginning of each training session, isokinetic training (multi-joint leg press and single joint knee extension) was performed in the Isomed 2000 (D&R Ferstl GmbH, Hemau, Germany). Exercise intensity was increased every 2 weeks as described in Table 1. The additional training consisted of a 2 day-split training program for the whole body. Each exercise was executed with the 8–10 repetition maximum (RM) with three sets and 1–2 min of inter-set recovery. Training loads were increased if necessary for every set to stay in the 8–10 RM range. All exercises are summarized in Table 1. The participants manually protocolled the additional training. The performance in the isokinetic training could automatically be saved with the training device and the mean power for each training session was calculated. In two training sessions, isokinetic training in leg extension could not be executed and was substituted by regular leg extension training. Technical problems with the isokinetic leg press forced the participants to train in a regular leg press in seven of the 24 training sessions. Therefore, only isokinetic training data of the single joint knee extension were analyzed.

**Table 1:** **Exercise description and progression**.

**Exercise and parameter**	**Weeks**			
	**1/2**	**3/4**	**5/6**	**7/8**
**LEG PRESS**				
Movement/Speed	CON: 200 mm/s	CON: 200 mm/s	CON: 150 mm/s	CON: 100mm/s
	ECC: 100 mm/s	ECC: 100 mm/s	ECC: 150 mm/s	ECC: 200mm/s
Repetitions	8	10	10	10
**LEG EXTENSION**				
Movement/Speed	CON: 45°/s	CON: 45°/s	CON: 60°/s	CON: 90°/s
	ECC: 90°/s	ECC: 90°/s	ECC: 60°/s	ECC: 45°/s
Repetitions	8	10	10	10
**ADDITIONAL EXERCISES**				
**Split 1**				
Abdominal twister	Bench press	External shoulder rotation	Abdominal flexion	Butterflies (cable cross)
Crunches	Barbell pullovers			
**Split 2**				
Back extensions and flies reverse	Barbell rows	Flies reverse cable cross	Lat pull	
Dumbell rows	Diagonal back extensions	Leg curls	Plank rotation	

*CON, concentric contractionz ECC, eccentric contraction*.

### Data acquisition and analysis

Data collection was kept identical for each measurement. MRI-scans of the quadriceps femoris were collected with a 1.5 T MRI scanner (Magnetom Avanto, Siemens Healthcare, Erlangen, Germany). Slices (5 mm slice thickness, T1 weighted, 0 mm interspace distance) were examined from L4 to the tibia tuberositas of the right leg and were analyzed by two experienced assessors. Regions of interest for each muscle in the M. quadriceps femoris were manually marked on every slice with a custom-designed Matlab program. For further analysis, the mean value of muscle volume for each operator was calculated for each slice and each muscle. Furthermore, based on this, muscle volume was calculated as the sum of anatomical cross-sectional area (ACSA) times slice thickness as described by Akagi et al. (2009).

Muscular strength was measured for the quadriceps muscle of the right leg. Resting twitch torques (RTT) were measured as described by Mau-Moeller et al. (2014). In short, participants were seated on a dynamometer (Isomed 2000, D&R Ferstl GmbH, Hemau, Germany) with 80° hip-flexion and 80° knee-flexion (0° = fully extended). No warm up was conducted before measurement to prevent twitch potentiation. Transcutaneous electrical femoral nerve stimulation was used to analyze the stimulus response curve. EMG-signals of the M. vastus medialis were recorded using bipolar EMG Ambu Blue Sensor N electrodes with 2 cm diameter. Maximal m-wave was recorded and five doublet (1 ms rectangular pulse, 400 V, 10 ms inter stimulus rest) stimulations were applied every 6 to 7 s (randomized) with 150% of maximal m-wave with a constant-current stimulator (Digitimer DS7A, Herfordshire, UK). Signals were amplified (×2,500), band-pass filtered (10–450 Hz) and digitized with a sampling frequency of 2 kHz through an analog-to-digital converter (DAQ Card TM -6024E; National Instruments, Austin, Texas, USA). Torque recordings were analyzed with a custom-designed Matlab program to calculate peak twitch torque (PTT), rate of torque development (RTD), and rate of torque relaxation (RTR). Interpolated twitches were analyzed as described by Behrens et al. (2014). In short, participants were seated in the same position as for RTT. Three maximally voluntary contractions were recorded with 2 min of rest between measurements. During contraction, a doublet stimulation was applied 1 s after torque onset. Additionally, a control twitch was applied 1–2 s (randomized) after torque was close to zero. Isometric maximum voluntary torque (iMVT), rate of voluntary torque development (RTD), and percent of voluntary activation (%VA) were calculated according to Strojnik and Komi (1998). For a better comparison, the data are presented as percent changes to the baseline-value measured before the first training session. As only two subjects were examined, no statistical analysis was performed.

## Results

### Training

Both participants were able to increase the training volume (sum of repetitions times training load). The weekly training volume for participant D increased by 43%, and for participant M by 50% during the 8-week training period. Mean isokinetic power per training session in the knee extensions also increased for both participants. Participant M increased power (P) by a mean of 21% (baseline: 101 W; P_max_: 151 W) with participant D increasing by a mean of 115% (baseline: 51 W; P_max_: 149 W) compared to the first training session. The prescribed training protocols for both participants required individual readjustment due to a hand injury of participant D in week four and reduced training capabilities of participant M due to muscle pain in the left thigh in week 7. The detailed time courses of both participants can be seen in Image 1 and Image 2 as Supplementary Material to this article. The complete dataset can be found as an electronic supplement to this article (Data Sheet 1) and a summary of the results can be found in Supplementary Table 2. A representative MRI scan for pre- and posttest can be found in Supplementary Image 3.

### Muscle volume

Participant D showed a higher increase of muscle volume during the training phase compared to participant M, with a maximum of 15.2 and 4.5%, respectively. Measured muscle volume of the M. quadriceps peaked in week 6 for subject D and in detraining week one for subject M. At the end of the training phase, both participants had higher muscle volumes than at the beginning of training. Participant D showed a rapid increase in volume after the first training week (6.2%), while participant M increased muscle volume only slightly with reasonable changes after 5 weeks of training (2.7%).

### Resting twitch torques

Peak twitch torque (PTT) decreased for both participants after the first week of training and stayed below baseline for participant D during the entire training period and at the end of the detraining phase (−6.7%). Participant M increased PTT in the detraining phase by 13.1% compared to baseline. TRTD (twitch rate of torque development) equally decreased after the first week of training and only increased in subject M after the detraining phase (5.3%). Rate of torque relaxation showed a similar time course as the other twitch parameters. In general, participant D had a higher decrease from baseline of PTT, TRTD, and TRTR (twitch rate of torque relaxation) compared to subject M.

### Voluntary activation and maximum strength

Isometric maximal voluntary torque (iMVT) increased rapidly between the pre-test and baseline for both participants. With the level of voluntary activation (VA) increasing 0.7 and 1.4% for participant D and M, respectively. The first 4 weeks of training were characterized by a slight decrease of iMVT and an increase of %VA. Then both participants increased their iMVT with slightly decreasing levels of %VA. iMVT decreased rapidly with thigh pain in subject M (week seven and eight), also being visual in the %VA data and the RTD.

## Discussion

### Results and interpretation

Our study shows that the adaptation of muscular parameters to intense strength training is a complex process. While muscle volume increased, net muscular strength—measured in the form of resting twitch torques—decreased and even stayed below baseline in one subject. As shown by Behrens et al. (2012), the influence of muscular damage reduces the force production in the twitch parameters for a long period of time. For the two analyzed participants, even after 8 weeks of training, strength parameters were still reduced compared to baseline. However, in the present study, the participants were training three times a week with high intensity compared to one stress-protocol in the study by Behrens et al. (2012). For both participants, values only returned to or exceeded baseline values in the detraining phase. This also suggests a considerable influence of fatigue on muscle force. The highest increase in strength gain can be seen from pre-test to baseline for the voluntary data, indicating learning effects to the test setup. During the training phase, both participants increased voluntary strength in some weeks, showing neuronal adaptation, as also confirmed by the increased voluntary activation level (%VA). Interestingly, %VA decreased after an initial increase in the first 4 weeks. A simultaneous increase of muscle volume and iMVT as described by DeFreitas et al. (2011) could not be found in the individual data sets. However, in the presented data, a high increase of muscle volume was found for the untrained participant after the first training week, as described by DeFreitas et al. (2011), possibly being explained by muscular edema. Participant M reported intense strength training before the beginning of the study. This could explain the lower decrease of twitch parameters and the lower increase of muscle volume with the start of the training phase when compared to participant D, who reported no prior strength training experience. If the measured volume directly reflected muscle volume and thus physiological cross sectional area (Alexander and Vernon, 1975), a parallel increase of muscle force would have been observable (Close, 1972; Narici et al., 1989).

For future studies, more participants should be included and a longer training period should be realized to account for strong changes in muscle volume, especially in untrained subjects. As participant D could not increase strength values compared to baseline, also the detraining phase should be prolonged in future studies. Additionally, other parameters have to be considered, if a complete description of adaptation was intended, including muscle fiber distribution, pennation angle, and fascicle length (Wisdom et al., 2014). From this pilot study we learnt that a method is needed to better interpret the degree of muscular edema, especially in the first part of the intervention. We will now include such a method in the thorough redesign of our protocol before expanding the study to more participants.

With such a methodological approach it will be possible to analyze muscular adaptation to intense strength training with a high level of detail. Controlled training as described in this study can generate a dataset for the validation of models for muscular adaptation.

### Limitations

As a limitation of the study, only two participants were examined due to the high costs of the measurements and the detailed measurement procedure. Therefore, no general conclusions can be drawn. Further, the measured values could have been influenced by a remaining fatigue from the weekly training session 2 days before measurement. Thus, the actual positive training effects can hardly be investigated with the presented setup. Additionally, nutrition and additional physical activities were not controlled for the participants.

## Ethics statement

Ethical committee of the medical faculty of the Eberhard-Karls-University of Tübingen. Both participants gave written and informed consent to the experimental procedure.

## Author contributions

All authors contributed to this article. Data was acquired and analyzed by NB, DB, JW, and DH. NB, DB, EK, WA, and SS created the conceptual design. All authors revised the work and have approved the final version. Further, all authors agree to be accountable for all aspects of the work.

### Conflict of interest statement

The authors declare that the research was conducted in the absence of any commercial or financial relationships that could be construed as a potential conflict of interest.

## Supplementary material

Supplementary Image 1 **Detailed time course of participant M**. Twitch parameters [peak twitch torque (PTT), twitch rate of torque development (TRTD), twitch rate of torque relaxation (TRTR)] are marked in gray, muscle volume of the quadriceps muscle in black, voluntary strength parameters [isometric maximum voluntary torque (iMVT), rate of voluntary torque development (RTD), % of voluntary activation (%VA)] are marked in orange. The bottom part of the figure shows the increases in training volume of the additional training and the development of isokinetic power in the leg extension exercises.

Supplementary Image 2 **Detailed time course of participant D**. Twitch parameters [peak twitch torque (PTT), twitch rate of torque development (TRTD), twitch rate of torque relaxation (TRTR)] are marked in gray, muscle volume of the quadriceps muscle in black, voluntary strength parameters [isometric maximum voluntary torque (iMVT), rate of voluntary torque development (RTD), % of voluntary activation (%VA)] are marked in orange. The bottom part of the figure shows the increases in training volume of the additional training and the development of isokinetic power in the leg extension exercises.

Supplementary Image 3 **Pre study (blue) and post detraining (yellow) muscle outlines of the quadriceps muscle of both subjects (left: D, right: M, one representative MRI slice)**.

Supplementary Table 2 **Periodic results of all analyzed parameters**. RF, M. rectus femoris volume; VL, M. vastus lateralis volume; VM, M. vastus medialis volume; VI, M. vastus intermedius volume; QC, M. quadriceps volume; PTT, peakt twitch torque; TRTD, twitch rate of torque development; TRTR, twitch rate of torque relaxation; iMVT, isometric maximal voluntary torque; RTD, isometric rate of torque development; %VA, level of voluntary activation.

Data Sheet 1 **Results virtual gym study**.
