# Supplementary figures and images for: Weekly Time Course of Neuro-Muscular Adaptation to Intensive Strength Training
Source: Front Physiol. 2017 Jun 8;8:329. doi: 10.3389/fphys.2017.00329 (PMC5462902; doi:10.3389/fphys.2017.00329)

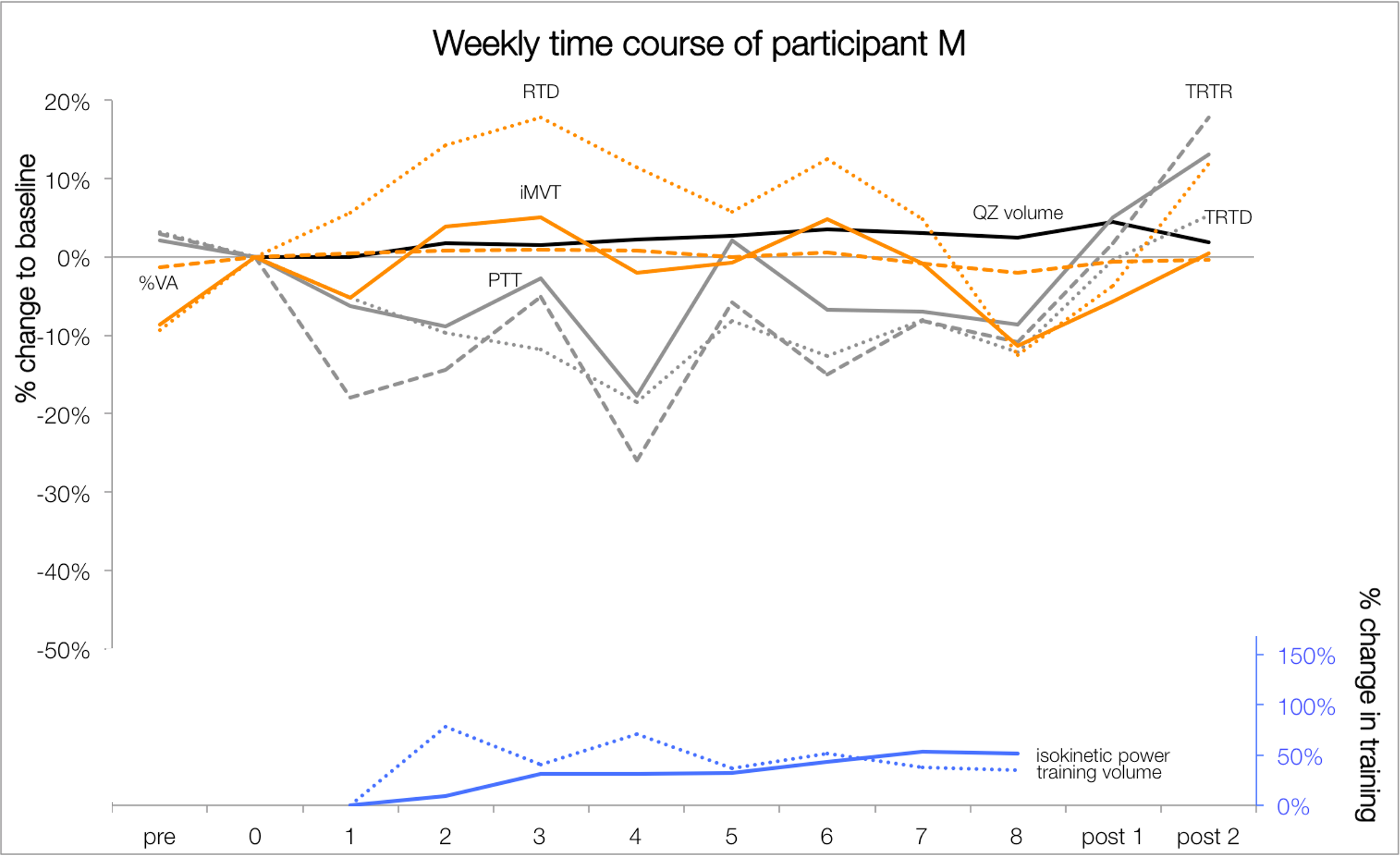

Supplement: Supplementary Image 1 — Detailed time course of participant M. Twitch parameters [peak twitch torque (PTT), twitch rate of torque development (TRTD), twitch rate of torque relaxation (TRTR)] are marked in gray, muscle volume of the quadriceps muscle in black, voluntary strength parameters [isometric maximum voluntary torque (iMVT), rate of voluntary torque development (RTD), % of voluntary activation (%VA)] are marked in orange. The bottom part of the figure shows the increases in training volume of the additional training and the development of isokinetic power in the leg extension exercises. [file Image1.TIFF]

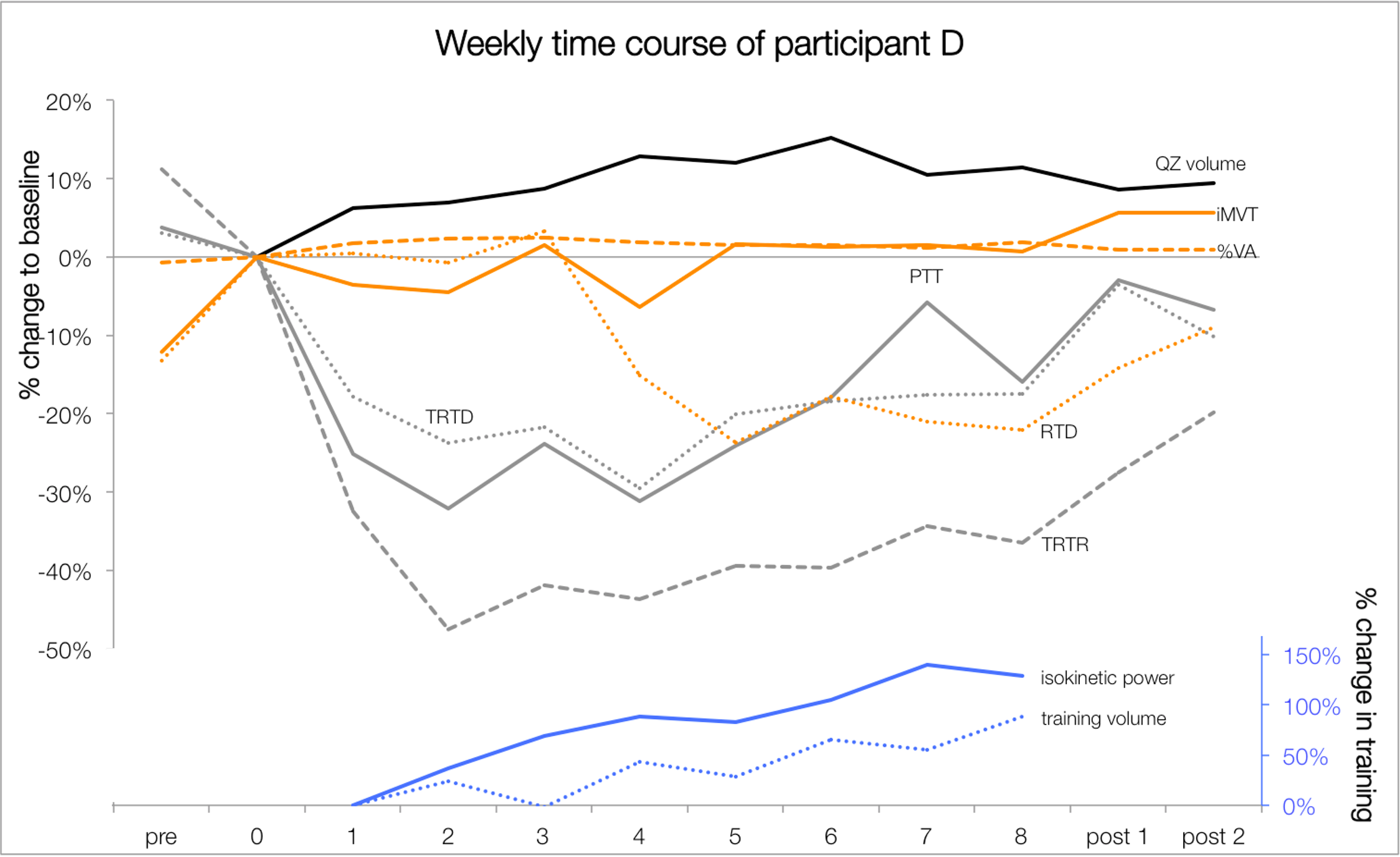

Supplement: Supplementary Image 2 — Detailed time course of participant D. Twitch parameters [peak twitch torque (PTT), twitch rate of torque development (TRTD), twitch rate of torque relaxation (TRTR)] are marked in gray, muscle volume of the quadriceps muscle in black, voluntary strength parameters [isometric maximum voluntary torque (iMVT), rate of voluntary torque development (RTD), % of voluntary activation (%VA)] are marked in orange. The bottom part of the figure shows the increases in training volume of the additional training and the development of isokinetic power in the leg extension exercises. [file Image2.TIFF]

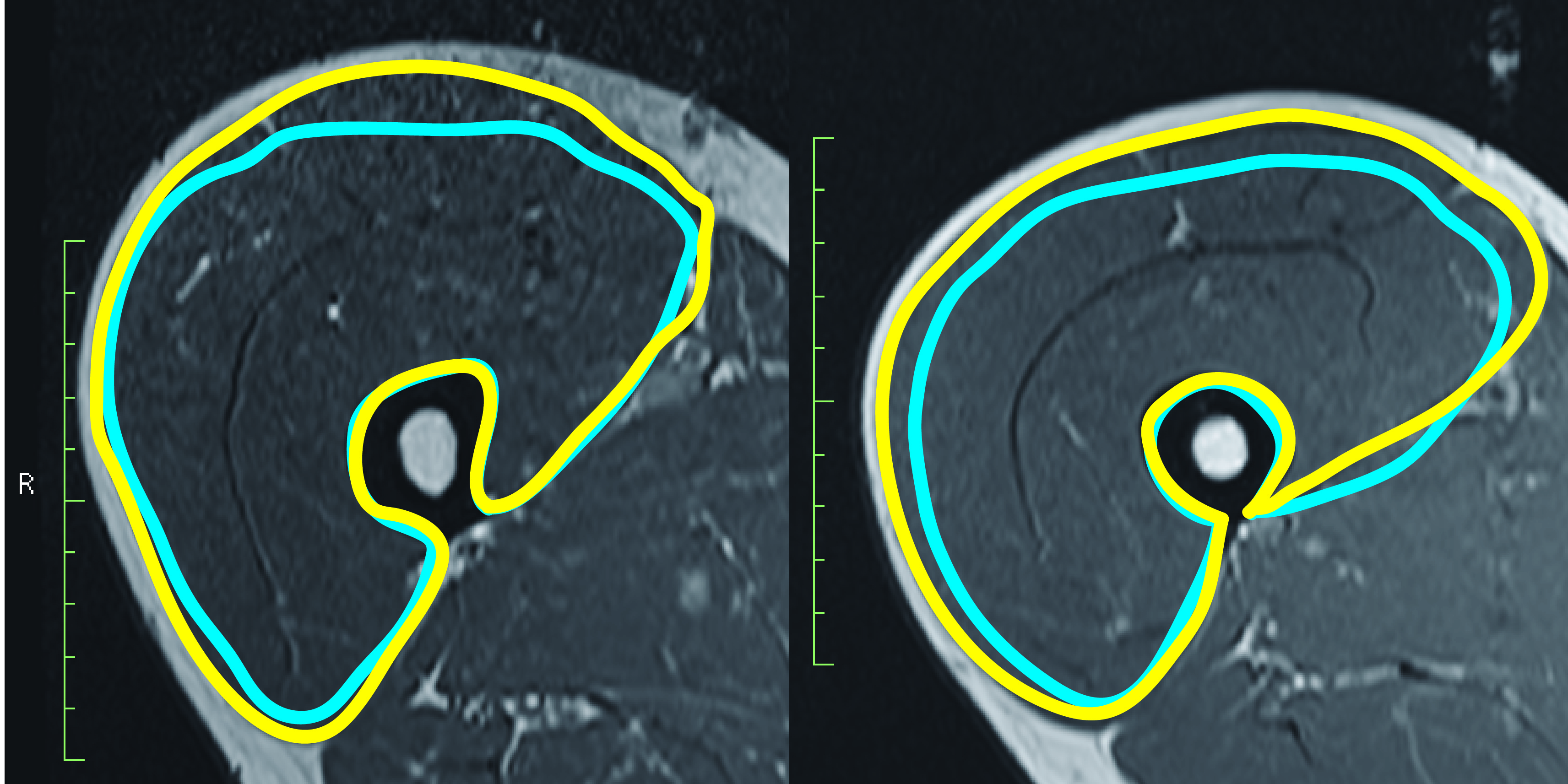

Supplement: Supplementary Image 3 — Pre study (blue) and post detraining (yellow) muscle outlines of the quadriceps muscle of both subjects (left: D, right: M, one representative MRI slice). [file Image3.jpg]
