# Supplementary material for: Weekly Time Course of Neuro-Muscular Adaptation to Intensive Strength Training
Source: Front Physiol. 2017 Jun 8;8:329. doi: 10.3389/fphys.2017.00329 (PMC5462902; doi:10.3389/fphys.2017.00329)
Supplement: Supplementary Table 2 — Periodic results of all analyzed parameters. RF, M. rectus femoris volume; VL, M. vastus lateralis volume; VM, M. vastus medialis volume; VI, M. vastus intermedius volume; QC, M. quadriceps volume; PTT, peakt twitch torque; TRTD, twitch rate of torque development; TRTR, twitch rate of torque relaxation; iMVT, isometric maximal voluntary torque; RTD, isometric rate of torque development; %VA, level of voluntary activation. [file Table2.DOCX]

Tab. 2 Periodic results of all analyzed parameters. RF=M. rectus femoris volume; VL=M. vastus lateralis volume; VM=M. vastus medialis volume; VI=M.vastus intermedius volume; QC=M. quadriceps volume; PTT= peakt twitch torque; TRTD=twitch rate of torque development; TRTR=twitch rate of torque relaxation; iMVT=isometric maximal voluntary torque; RTD=isometric rate of torque development; %VA=level of voluntary activation.

| subject | parameter | baseline | after 4 weeks | after 8 weeks | after detraining |
| --- | --- | --- | --- | --- | --- |
| D | QC (cm^3^) | 2361.5 | 2664.9 | 2631.4 | 2583.2 |
| M |  | 2294.3 | 2345.2 | 2350.1 | 2337.9 |
| D | PTT (Nm) | 107.4 | 73.8 | 90.3 | 100.2 |
| M |  | 84.4 | 69.4 | 77.1 | 95.4 |
| D | TRTD (Nm/s) | 2649.7 | 1866.0 | 2187.4 | 2380.0 |
| M |  | 2269.8 | 1847.8 | 1993.3 | 2389.6 |
| D | TRTR (Nm/s) | -1385.7 | -779.9 | -879.8 | -1111.2 |
| M |  | -1025.2 | -758.3 | -913.6 | -1208.2 |
| D | iMVT (Nm) | 316.9 | 296.7 | 319.0 | 334.9 |
| M |  | 281.5 | 275.9 | 249.5 | 282.7 |
| D | RTD (Nm/s) | 2116.1 | 1797.2 | 1648.8 | 1925.1 |
| M |  | 1786.0 | 1990.2 | 1562.6 | 1998.6 |
| D | %VA (%) | 96.7 | 98.6 | 98.5 | 97.7 |
| M |  | 98.2 | 98.9 | 96.1 | 97.7 |
